# How Much Orthodontia Should the General Practitioner Do?

**Published:** 1905-08-15

**Authors:** Martin Dewey

**Affiliations:** Grand Rapids, Mich.


### HOW MUCH ORTHODONTIA SHOULD THE GENERAL PRACTITIONER DO?

BY MARTIN DEWEY, D.D.S., M.D., GRAND RAPIDS, MICH.

An Address delivered before the Southwestern Michigan Dental Society, April 11 and 12, 1905.

The practice of orthodontia is by no means new, as we find the first text books on dentistry contained chapters on "the correction of irregularities of the teeth," but it is within recent years that we find interest has increased to such an extent that any dental journal you may pick up almost invariably contains articles on orthodontia. The cause of this new and increased interest in the subject of orthodontia is the fact that orthodontia has established itself as a *specialty of dentistry.* No one thing has done more toward establishing orthodontia as a specialty than the organization of the American Society of Orthodontists.

When the society was first organized the "wise-acres" of dentistry said the society could not exist because orthodontia was too limited a subject to support a society of its own. The growth of the society has been steadily onward and upward until we find, at the recent International Dental Congress, the section of orthodontia was the most successful of them all.

Since orthodontia has established itself as a *specialty of dentistry* the question naturally follows, "What will be the attitude of the Dental Profession toward Orthodontia in its proper sphere?" Will the profession continue to do orthodontia in the future as it *has* in the past and *is doing* in the present, or will it confine itself to operations in general dentistry for which it is better fitted?

Whatever the attitude of the dental profession may be, it will necessarily be governed by the attitude of the individual practitioner. In other words, "How much Orthodontia should the General Practitioner do?" To answer this question in a few words, I will say, "Not any." My reason for answering the question in this manner is the fact that orthodontia has been and is yet a neglected subject in the dental college curriculum; lectures on this subject are generally given by some one occupying some other chair in the college. Those lectures are generally on, "How to construct regulating appliances," "the taking of modeling compound impressions" "the extraction of teeth for the correction of mal-occlusion" and "when and what teeth to extract." The result of such teaching has been the extraction of any tooth from the third molar to the central incisor. We still find prominent men, high in the profession of dentistry, advocating the extraction of the first permanent molar for the *correction* of mal-occlusion of the anterior teeth. I will say the case never did exist in which the occlusion of the anterior teeth was benefited by the extraction of the first permanent molar; not only was there failure to benefit the existing mal-occlusion, but the patient's *facial outline was spoiled* and the *condition complicated* by the production of a *mal-occlusion* of the *molar region*.

We also find the theory of inherited mal-occlusions is still taught by some men. They claim that the "intermarriage of types," "mixing of races" and the "inheritance of large teeth and small jaws" cause a large percentage of mal-occlusions. The theory in itself is faulty because the tendency of nature is to harmonize conditions. If we saw such a deformity existing in the teeth and jaws, why would not a like condition exist in other parts of the anatomy? For example, why would you not see large fingers on small hands or large finger nails on small fingers? If the child would inherit large teeth and small jaws why would you not see equally many cases of small teeth and large jaws? But we find the result of this teaching is, that dentists have practiced the extraction of teeth for the correction of mal-occlusions, claiming that the case could not be otherwise treated because of this inherited condition. We even find men have gone so far as to extract some of the anterior teeth for the correction of mal-occlusion justifying their acts by claiming the teeth were too large for the arches. Consequently a great deal of harm is still being done by those who are attempting to correct mal-occlusions by extraction. Not only do they fail to obtain normal occlusions, but the condition is often made worse from the standpoint of an ideal arrangement of the teeth, and they furthermore produce harm by spoiling the facial outline of the patient. It is the result of seeing so *many of these failures*, the result of the *harm* that is being done, and the needless

*sacrifice* of teeth that has caused me to say that the general practitioner should abandon the practice of orhtodontia as a general rule.

Another reason why the general practitioner should confine himself to the practice of general dentistry is the fact that it is impossible to successfully practice orthodontia in connection with another practice. Like oil and water, they refuse to mix. He will either neglect his general practice or his orthodontia patients. He may set aside certain hours of the day and faithfully promise himself that he will do nothing but orthodontia during that hour but the first thing he knows his general practice has crowded into the time set aside for the treatment of cases of mal-occlusions. He will have a patient in the chair, making a gold filling when the orthodontia patient comes in. He cannot ask the patient to sit in the chair with the rubber dam on while he waits on the patient with the mal-occlusion. He tells the orthodontia patient to come back to-morrow, but to-morrow the patient has a ticket to the matinee or other social engagement so the appointment is lost entirely. The result of this is that the dentist becomes discouraged and so the appliance is removed before the case is completed and another failure is recorded. It is also impossible for the general practitioner to acquire the same technical skill by devoting a few hours each day to the practice of orthodontia that a specialist would by devoting his entire time to it. The general practitioner may possess a good knowledge of modern orthodontia, but unless he possesses the technical knowledge his operations will necessarily be second class. You have no right to ask your patients to be content with operations in orthodontia which are not the best any more than you have the right to ask them to be content with second class work in operative dentistry and crown and bridge work. The general practitioner, not possessing the required skill, will necessarily have to treat cases at a financial loss. It would be dollars and cents to his advantage if he would confine himself to the field of general dentistry and recommend patients in need of orthodontia treatment to one who devotes his entire time to the treatment of mal-occlusions.

But the usefulness of the general practitioner in orthodontia still exists. On him depends to a great extent the rapidity with which orthodontia will develop as a specialty. His usefulness to orthodontia in the future will be that of advisor. It will be he, who will first see the patients suffering from mal-occlusions, and in order that he may give good advise he must necessarily be familiar with the teachings of modern orthodontia. He must be familiar with normal occlusion, recognize the relation the teeth of one arch bear to the other, also be familiar with the laws of normal occlusion. He must be familiar with the temporary teeth as possible factors in the production or prevention of mal-occlusion in the permanent teeth. He must also recognize patients suffering from nasal obstructions, advise early treatment of the difficulty so as to obtain the best results from the standpoint of occlusion and facial art. When the dentist becomes familiar with the principles of modern orthodontia we will see fewer failures such as the treatment of cases in which the mal-occlusions of the upper arch have been corrected without any consideration being taken of the lower arch. The question may be raised that the general practitioner is forced to do some orthodontia because the specialists are too few. Whenever the dental profession shows a greater tendency toward recommending patients suffering from mal-occlusions to men devoting their entire time to the treatment of such cases, specialists will become more numerous and until that time the general practitioner who attempts to do orthodontia must be sure that anything he does will not be of such nature as to produce harm in after years. He must not forget that the so-called simple case of mal-occlusion in which he sees only a "crooked tooth" is but the symptom of some greater trouble. That a tooth crowded out of the arch is but the symptom of a greater mal-occlusion. He should avoid extraction for the correction of mal-occlusions and all cases which he treats must be toward the high ideal of normal occlusion and improved facial outlines, and when he has attained these results he has obtained the best results possible in modern orthodontia.

